# Relationships of self-management abilities to loneliness among older people: a cross-sectional study

**DOI:** 10.1186/s12877-020-01584-x

**Published:** 2020-05-27

**Authors:** Anna Petra Nieboer, KlaasJan Hajema, Jane Murray Cramm

**Affiliations:** 1 Erasmus School of Health Policy & Management, Erasmus University Rotterdam, Burgemeester Oudlaan 50, 3062 PA Rotterdam, The Netherlands; 2 Public Health Service Zuid Limburg, Academic Collaborative Centre for Public Health Limburg, Heerlen, The Netherlands

**Keywords:** Loneliness, Aging, Self-management, Older people

## Abstract

**Background:**

We investigated relationships of broader self-management abilities (self-efficacy, positive frame of mind, investment behavior, taking initiatives, multifunctionality of resources, variety of resources) to social and emotional loneliness among community-dwelling older people while controlling for background characteristics.

**Methods:**

This cross-sectional study employed a representative sample of 41,327 community-dwelling people aged ≥55 years in Limburg, the Netherlands, identified using the population register (weighted per district, complex sampling design). In total, 20,327 (50%) people responded to the questionnaire.

**Results:**

All self-management abilities were associated negatively with emotional loneliness. Taking initiatives, multifunctionality, self-efficacy, and a positive frame of mind were associated negatively with social loneliness. Self-efficacy had the strongest relationships with social and emotional loneliness.

**Conclusions:**

In combatting loneliness among older people, investment in their ability to self-manage their social lives and activities, such as increasing opportunities for positive social interaction and social support and reducing maladaptive cognition, seems to be crucial.

## Background

The Netherlands — along with the rest of the world — faces serious challenges as a direct consequence of populational aging. The absolute number of older people in the Netherlands is expected to increase from 2.4 million (16%) in 2010 to 4.6 million (26%) in 2040 [1, 2]. The same picture emerges across Europe, where the percentage of people aged ≥65 years increased from 13.7% in 1990 to 17.4% in 2010, and is predicted to increase even further to 30% by 2060 [3]. Loneliness is highly prevalent among older people [4–6]; up to one-third of older people experience feelings of loneliness [7–9]. Research clearly shows the detrimental impacts of loneliness on health outcomes, morbidity, mortality, and healthcare use [10–15].

Although loneliness is especially prevalent among the very old, it is not related to age alone [4, 16]. Associations between age and loneliness can be explained mainly by age-related health problems and changes in social network ties [17–22]. Societal changes in recent decades, such as the reduction of family size, increased number of people who stay single throughout their lives, increased divorce rates, greater distances between family members, and reduced number of people living in multigenerational households, have influenced the occurrence of social and emotional loneliness among older people [4, 23]. Furthermore, associations have been found between loneliness and older people’s background characteristics. Marital status, for example, is clearly associated with loneliness, with married people found to be at an advantage in this regard in many studies [17–19, 22, 24, 25]. Mixed findings, however, have been reported on the relationship between gender and loneliness. Although women appear to be lonelier than their male counterparts [26], this gender effect disappears after adjustment for socioeconomic and health variables [19, 27, 28]. Mixed results have also been obtained regarding the relationship between loneliness and socioeconomic status (income and education); some researchers have reported such an association [9, 21, 25], whereas others have found none [19, 29, 30].

Perlman and Peplau [31], p. 31 defined loneliness as “the unpleasant experience that occurs when a person’s network of social relationships is deficient in some important way, either quantitatively or qualitatively.” Loneliness occurs when the number of existing relationships a person has is smaller than desired (quantitative deficiency in one’s network of social relationships) or when the desired intimacy has not been realized (qualitative deficiency in one’s network of social relationships) [32, 33]. It is an expression of negative feelings about certain missing aspects in a person’s social relationships which can occur throughout the life-course [33]. The prevalence is, however, much higher among individuals of advanced age [8, 9]. Weiss [34] identified two components of loneliness: emotional loneliness (stems from the absence or loss of a close attachment relationship) and social loneliness (stems from the absence of an engaging social network). Emotional loneliness arises after life events, such as losing a loved one through death or ending of a marriage, leading to intense feelings of abandonment and emptiness. Social loneliness occurs, for example, when a person moves to a new place [33].

Knowledge about ways in which loneliness can be prevented and how feelings of loneliness can be reduced in older populations is urgently needed. Promising intervention strategies to reduce loneliness attempt to correct deficits in social skills, social support, opportunities for social interaction, and/or maladaptive social cognition [35–39]. Broader self-management abilities to maintain overall well-being could be considered to be important self-regulatory skills to handle these deficits and to combat loneliness in older populations. Steverink and colleagues [40] identified six self-management abilities: self-efficacy, positive frame of mind, investment behavior, taking initiatives, multifunctionality of resources, and variety of resources. Self-efficacy was originally defined by Bandura [41] and refers to the ability to gain and maintain a belief in one’s personal competence to achieve certain goals in life, such as reducing feelings of social and emotional loneliness. A person with stronger self-efficacy beliefs is more likely to undertake the activities and efforts needed to achieve such goals [40, 42]. According to Steverink and colleagues [40], having a positive frame of mind entails the ability to adopt and maintain a positive frame of mind or positive expectations, even after an illness or other major life event. The ability to stay positive despite certain challenging life events is expected to prevent feelings of loneliness because it extends the time horizon and boosts confidence, which encourages a person to engage in activities [40], and in turn may help prevent or reduce the worsening of feelings of loneliness. Investment behavior has proven to be crucial for achieving resource stability for older people [40, 42]. Without it, a (stronger) decline in resources is expected to increase the risks of social and emotional loneliness. Initiative taking refers to the ability to be instrumental or self-motivating, which is expected to prevent feelings of loneliness among older people; older people who often take initiatives are expected to report lower levels of loneliness compared with those who seldom take initiatives. The ability to gain and maintain a variety of resources entails having more than one resource or activity available to achieve a certain goal in life; for example, affection can be obtained from children, a partner, and a dear friend [40, 43]. Older people holding a variety of resources are expected to report higher levels of social and emotional well-being than are older people lacking such variety. Finally, multifunctional resources are resources and activities that serve multiple purposes simultaneously and in a mutually reinforcing way [40, 43], and are thus of special importance among older people who are experiencing a reduction of resources.

Although multiple studies have investigated the relationships between these six self-management abilities and well-being in older populations (e.g., [44–46]), we found few such studies that considered loneliness; one study investigated loneliness and the self-management abilities of self-efficacy and taking initiatives in visually impaired elderly people, and an intervention study developed by Steverink and colleagues incorporated all six self-management abilities and was conducted with older women [47, 48]. We currently lack research on the relationships between all six self-management abilities and social and emotional loneliness in a large representative sample of older persons. Given that loneliness is an urgent issue, especially in older populations, such research is crucial. This study thus aimed to investigate the relationships between the six self-management abilities and social and emotional loneliness among community-dwelling older people while controlling for background characteristics.

## Methods

### Study design and population

This cross-sectional study was conducted with a representative sample of community-dwelling older people in Limburg (a region in the southern Netherlands). The study population was selected from respondents to the Health Monitor survey, for which the Community Health Service sends an extensive general health questionnaire by mail every 4 years to a large national sample of community-dwelling people [49, 50]. In Limburg, the theme of self-management was added to this questionnaire, which could be filled in online or on paper. In 2016, questionnaires were sent to 41,327 people aged ≥55 years who were identified using the population register (weighted per district and using a complex sampling design). Selection from this sample was random for all represented age groups; people living in neighborhoods with low socioeconomic status were overrepresented. Two reminders were sent to non-responders, and the response rate was 50% (*n* = 20,327). On review, the medical ethics committee of the Amsterdam University Medical Center determined that the rules laid down in the Medical Research Involving Human Subjects Act did not apply to the Health Monitor study, and thus waived the need for further ethical approval. Via a contract between the Erasmus University Rotterdam and the Community Health Service South Limburg, Jane Murray Cramm and Anna Petra Nieboer obtained permission to use the data collected by the Community Health Service South Limburg.

### Measures

#### Loneliness

We assessed social and emotional loneliness with the 11-item loneliness scale developed by De Jong Gierveld and Kamphuis [51]. Example items for the assessment of social loneliness are: “There are plenty of people I can rely on when I have problems” and “There are enough people I feel close to.” Examples of items used to assess emotional loneliness are: “I experience a general sense of emptiness” and “I often feel rejected.” Response categories are “yes,” “more or less,” and “no.” Item scores were dichotomized in agreement with the scaling procedure, with the response “more or less” indicating loneliness. Loneliness subscale scores were computed as the sums of dichotomized item responses, ranging from 0 (absence of emotional loneliness) to 6 (extreme emotional loneliness) for emotional loneliness and from 0 (not socially lonely) to 5 (extremely socially lonely) for social loneliness. The two scales have been proven to be reliable and valid for the assessment of social and emotional loneliness, respectively [21].

#### Self-management abilities

We measured older people’s self-management abilities with the Self-Management Ability Scale Short version (SMAS-S) [42]. This instrument measures the six self-management abilities of self-efficacy, positive frame of mind, investment behavior, taking initiatives, multifunctionality of resources, and variety of resources. Average SMAS-S scores range from 1 to 6, with higher scores indicating better self-management abilities. Cramm and colleagues [42] showed that the psychometric properties of the SMAS-S are good and that it is valid and reliable for the assessment of self-management abilities in older populations.

#### Background characteristics

We also asked respondents about their age, gender, marital status (single or not), and educational level [low (lower-level applied education), medium (medium-level applied education), or high (high-level education preparing for applied science or research university)]. We inquired whether they were chronically ill.

### Data analysis

The data were analyzed using IBM SPSS Statistics 25.0 software (IBM Corporation, Armonk, NY, USA). The data were weighted to generate appropriate population estimates using a complex sampling design. Weighted percentages, means, and standard errors of the study variables were used in the reporting of study results. To identify relationships of background characteristics and self-management abilities to social and emotional loneliness, a general linear model with a complex samples design was employed for regression analyses (given the large sample, we used 0.01 instead of 0.05 as the significance level, default listwise deletion of missing cases was used).

## Results

Table 1 displays descriptive statistics for the study population. Respondents’ mean age was 67.96 years, about half (47.9%) of them were male, and the majority (71.9%) were married. Mean social and emotional loneliness scores were 2.03 [standard error (SE) 0.2, range 0–5] and 1.51 (SE 0.02, range 0–6), respectively. Among self-management abilities, the lowest score was for the variety of resources (mean 3.66, SE 0.01) and the highest score was for investment behavior (mean 4.58, SE 0.01).

**Table 1:** Background characteristics and self-management ability and loneliness scale scores for the study population

Characteristic	Mean (SE) or frequency	n
Age (years)	67.96 (0.05)	20,742
Gender (male)	47.9%	20,742
Marital status (married)	71.9%	20,442
Educational level		19,088
Low	53.4%	
Medium	25.3%	
High	21.3%	
No chronic illness/condition	47.9%	20,475
SMAS-S scores		
Taking initiatives	4.31 (0.01) range 1–6	19,961
Investment behavior	4.58 (0.01) range 1–6	19,937
Variety of resources	3.66 (0.01) range 1–6	19,822
Multifunctionality	4.04 (0.01) range 1–6	19,902
Self-efficacy	4.27 (0.01) range 1–6	19,941
Positive frame of mind	4.02 (0.01) range 1–6	19,943
Loneliness scale scores		
Emotional loneliness	1.51 (0.02) range 0–6	18,758
Social loneliness	2.03 (0.02) range 0–5	18,982

*SMAS-S* Self-Management Ability Scale Short version

In multivariate regression analyses corrected for background variables, all six self-management abilities were associated negatively with emotional loneliness, indicating that they protect against feelings of emotional loneliness among older people (Table 2). The strongest relationship was found between emotional loneliness and self-efficacy (β = − 0.64). Taking initiatives, multifunctionality, self-efficacy, and positive frame of mind were associated negatively with social loneliness, indicating that they protect against feelings of social loneliness among older people. The strongest relationship was found with self-efficacy (β = − 0.56).

**Table 2:** Relationships of background characteristics and self-management abilities to loneliness, as determined by regression analyses

Variable	Emotional loneliness *n* = 17,175			Social loneliness *n* = 17,342		
	β	SE	*p*	β	SE	*p*
Intercept	6.78	0.22	< 0.001	7.14	0.18	< 0.001
Age	−0.00	0.00	0.643	− 0.01	0.00	< 0.001
Gender (male)	−0.15	0.03	< 0.001	0.29	0.03	< 0.001
Marital status (married)	− 0.99	0.04	< 0.001	−0.27	0.04	< 0.001
Low educational level^a^	0.03	0.04	0.511	−0.04	0.04	0.337
Medium educational level^a^	−0.02	0.05	0.715	0.06	0.04	0.161
No chronic illness/condition	−0.41	0.03	< 0.001	−0.19	0.03	< 0.001
Taking initiatives	−0.09	0.03	0.006	−0.23	0.03	< 0.001
Investment behavior	−0.21	0.04	< 0.001	−0.02	0.03	0.518
Variety of resources	−0.10	0.02	< 0.001	0.02	0.02	0.446
Multifunctionality	−0.13	0.03	< 0.001	−0.11	0.02	< 0.001
Self-efficacy	−0.64	0.03	< 0.001	−0.56	0.03	< 0.001
Positive frame of mind	−0.06	0.02	0.002	−0.05	0.02	0.019
*R*^2^	0.29			0.23		

^a^Reference group = high educational level

## Discussion

This research aimed to investigate relationships of broader self-management abilities to social and emotional loneliness among community-dwelling older people, while controlling for background characteristics. The results clearly show the importance of consideration of these abilities in efforts to understand loneliness in older populations.

All six self-management abilities were associated negatively with emotional loneliness, and four of these abilities were associated with social loneliness; self-efficacy had the strongest relationship with both forms of loneliness. These findings are in line with those of Alma and colleagues [52], who showed that self-efficacy and loneliness are related strongly in visually impaired elderly persons, and those of Fry and Debats [29], who showed that self-efficacy beliefs were a significantly stronger predictor of loneliness than were sociodemographic characteristics. These findings are important in a time of aging populations and high prevalence of social and emotional loneliness [8, 9]. Whereas earlier studies revealed relationships between loneliness and background characteristics (e.g., age, gender, marital status, educational level), this research adds to our knowledge by showing that self-management abilities remain associated with older people’s social and emotional loneliness after controlling for these background characteristics.

People’s social networks are known to contract, likely leading to a lack of quality relationships [32], as they grow older. Older people with stronger self-efficacy beliefs and positive and self-supportive thoughts, who actively invest in their social relationships, are better able to prevent feelings of loneliness than are older people with poorer self-management abilities. Older people who lack such skills communicate less with their neighbors and are less likely to seek help from others [53], which increases feelings of social and emotional loneliness, as well as the risk of social isolation. Investment in older people’s self-management abilities may help them to continue investing in their existing social relationships, and to find ways to create new relationships, which would help to prevent feelings of social loneliness. However, the avoidance of loneliness it is not simply a matter of the presence of others, but rather the presence of others who value one, whom one can trust, and with whom one can communicate [54, 55].

Older people with better self-management abilities are probably also better able to create the support system they need, including emotional, informational, and instrumental support, which prevents feelings of loneliness [56]. Cattan and colleagues [57] concluded in their review of intervention studies that the programs that reduced loneliness most effectively were group interventions. The organization of regular group meetings is a way to increase the number of one’s friends [58]. Moreover, educational and social activity interventions targeting specific groups can reduce social isolation in older adults [35, 57]. However, interventions should not focus on social relations alone [38]; more than one intervention strategy, including those that address maladaptive social cognition and enhance social support [38, 55], should be used. In accordance with these findings, Kremers and colleagues [47] reported improvement in self-management ability and social and emotional loneliness among single women aged ≥55 years participating in a self-management group intervention based on the self-management of well-being theory. The six self-management abilities defined by Steverink et al. [40] help older people to manage their resources in such a way that their overall well-being is maintained or even improved, and losses are avoided or coped with adequately. These behavioral and cognitive self-management abilities can be addressed in interventions [48] and improve social skills, social support, opportunities for social interaction, and/or maladaptive social cognition. Given their associations with social and emotional loneliness in the current study, this approach would be promising, especially among older persons with long-established and late-onset loneliness [9].

Although the heterogeneity of the tools used to measure loneliness prohibits direct comparison among studies, evidence suggests that the prevalence of “some degree of loneliness” [7], but not that of “persistent loneliness” (feeling lonely often, mostly, or always during the past week) [4], increased in recent decades. Indirect estimates of social and emotional loneliness, such as those based on De Jong-Gierveld and Kamphuis’ loneliness scale [51], do not increase over time, except for participants with activity limitations [16], and even decreased slightly among 64–84-year-old women in one study [6]. The variability of loneliness in later life calls for appropriately diverse interventions [9].

We distinguished between social and emotional loneliness on theoretical grounds [34], although empirically these concepts are highly correlated [38]. Dahlberg and McKee [59] showed that social and emotional loneliness are related but divergent constructs, with partially differing predictors. In the current study, taking initiatives, multifunctionality, self-efficacy, and a positive frame of mind were all important for both social and emotional loneliness, but investment behavior and the variety of resources were related only to emotional loneliness in multivariate analyses. Apparently, investment in an intimate relationship or close emotional attachment and the ability to gain and maintain a variety of resources to obtain affection protect against emotional, but not social, loneliness.

Several limitations of this study should be taken into account. First, the cross-sectional nature of the study prevents us from (1) showing how loneliness develops overtime (if it is stable or increasing) and (2) drawing causal conclusions. While our study showed that self-management abilities affect feelings of loneliness the relationship is dynamic; older people feeling lonely might view the world through “blue-colored glasses,” feeling less efficacious, have a less positive frame of mind, be less instrumental and/or less motivating. Both emotional and social loneliness are subjective appraisals which make the dynamic relationship plausible. Second, we found that self-management abilities are related to social and emotional loneliness among older people, but we did not investigate how these abilities can be improved nor did we look at age-related differences in activity patterns. Although promising interventions are currently available their longitudinal effects are still largely unknown. The large sample is a strength of this study, as is its representativeness of people aged 55 years or older in the province of Limburg, the Netherlands. Life expectancy in the Netherlands for men is (78.9) and women (83.2) [60].

## Conclusions

In combatting loneliness among older people, investment in their ability to self-manage their social lives and activities, such as through regular socialization with family, friends and neighbors, seems to be crucial. Interventions that aim to enhance the self-management abilities of self-efficacy, positive frame of mind, investment behavior, taking initiatives, multifunctionality of resources, and variety of resources are expected to be useful additions to current public health interventions.

## Data Availability

Via a contract between the Erasmus University Rotterdam and the Community Health Service South Limburg, Jane Murray Cramm and Anna Petra Nieboer obtained permission to use the data collected by the Community Health Service South Limburg. The datasets analysed during the current study are available from the corresponding author on reasonable request.

## Abbreviation

SMAS-S: Self-Management Ability Scale Short version
